# Electrophysiological properties of melanin-concentrating hormone neuron subpopulations defined by anatomical localization and CART expression

**DOI:** 10.3389/fncel.2024.1439752

**Published:** 2025-01-22

**Authors:** Rafiat Damilola Adekunle, Mohammed Sohel Chowdhury, Lisa Z. Fang, Michiru Hirasawa

**Affiliations:** Division of Biomedical Sciences, Faculty of Medicine, Memorial University, St. John’s, NL, Canada

**Keywords:** melanin-concentrating hormone (MCH), intrinsic excitability, cocaine and amphetamine-regulated transcript (CART), hypothalamus, zona incerta, H-current, whole cell patch clamp

## Abstract

**Introduction:**

Melanin-concentrating hormone (MCH) neurons are essential regulators of energy and glucose homeostasis, sleep–wake behaviors, motivation, learning and memory. These neurons are anatomically distributed across the medial (MH) and lateral hypothalamus (LH), and the adjacent zona incerta (ZI), which may represent functional subgroups with distinct connectivity with different brain regions. Furthermore, MCH neurons can be classified according to co-expression of neuropeptides, such as cocaine and amphetamine- regulated transcript (CART).

**Methods:**

To identify functional similarities and differences of MCH subpopulations, we characterized their intrinsic electrophysiological properties using whole cell current clamp recording on acute brain slices from male and female mice.

**Results:**

MCH neurons were classified into subgroups according to their anatomical localization in three MCH-rich brain areas: MH, LH and ZI. Among the three brain regions, ZI MCH neurons were the least excitable while LH MCH neurons were the most excitable. Furthermore, grouping MCH neurons according to CART co-expression revealed that MCH/CART− cells are uniquely depolarized and excitable, and display H-currents. These MCH/CART− cells were mainly found in the LH, which may in part explain why LH MCH neurons are more excitable. While some sex differences were found, the majority of parameters investigated were not different.

**Discussion:**

Our results suggest that MCH/CART− cells are electrophysiologically distinct, whereas MCH/CART+ cells are largely similar despite their diffuse distribution in the hypothalamus. It is therefore a combination of intrinsic electrophysiological properties and neurochemical identities, in addition to anatomy and connectivity that are likely to be critical in defining functional subpopulations of MCH neurons.

## Introduction

Melanin-concentrating hormone (MCH) is a 17-amino acid peptide that is expressed in the hypothalamus of the mammalian brain (Bittencourt, 2011). MCH-expressing neurons are distributed extensively within the hypothalamus, with particularly high density in the lateral hypothalamus (LH), the medial hypothalamus (MH), as well as the adjacent zona incerta (ZI). MCH neurons also project widely throughout the brain in a pattern consistent with the MCH1 receptor expression (Bittencourt et al., 1992; Chee et al., 2013), and these broad projections are thought to imbue MCH neurons with the ability to regulate many physiological functions including energy and glucose homeostasis, sleep, motivated behavior, memory, and mood (Qu et al., 1996; Borowsky et al., 2002; Alon and Friedman, 2006; Adamantidis et al., 2008; Kong et al., 2010; Izawa et al., 2022; Subramanian et al., 2023).

To underscore the wide range of physiological functions in which MCH neurons are involved, recent research has identified subpopulations of MCH neurons with distinct anatomical and/or neurochemical properties. A topographical organization of MCH neurons based on specific co-expressed neurochemicals (Fujita et al., 2021; Miller et al., 2024) or projection targets has been described (Chometton et al., 2014; Haemmerle et al., 2015). For example, MCH neurons that co-express cocaine and amphetamine-regulated transcript (MCH/CART+) are found predominantly in the MH and ZI, while MCH/CART− neurons are concentrated in the LH (Fujita et al., 2021; Wang et al., 2021; Miller et al., 2024). MCH/CART− neurons preferentially project toward the spinal cord, while MCH/CART+ neurons mainly send ascending inputs to innervate the cerebral cortex and medial septal complex, although some species differences have been reported (Cvetkovic et al., 2004; Croizier et al., 2010). In addition, the pattern of axonal inputs to the hypothalamus and ZI are also known to be specific to the source of projections (Fujita et al., 2021; Wang et al., 2021). Thus, MCH neurons found in different nuclei such as the LH, MH and ZI may constitute separate functional groups with distinct connectivity.

Previous studies have characterized the electrophysiological properties of MCH subpopulations subdivided by their neurochemical phenotype. These studies found differences in active and passive properties such as input resistance, action potential waveform, spike adaptation and excitatory synaptic properties between MCH neurons with or without CART expression (Fujita et al., 2021; Miller et al., 2024). Furthermore, some of these properties differ in a sex-dependent manner (Miller et al., 2024). Here, in an effort to harmonize the findings in the field, we characterized the electrophysiological properties of anatomical and neurochemical subpopulations of MCH neurons using whole-cell patch clamp. Our results add to the current understanding of the electrophysiological properties that define subpopulations of MCH neurons. These differences may be important in shaping specific patterns of activity necessary to produce certain functional outcomes.

## Materials and methods

### Animals

All animal experiments followed the guidelines of the Canadian Council on Animal Care and were approved by Memorial University Institutional Animal Care Committee (Protocol number 18-02-MH). C57BL/6NCrl mice were obtained from Charles River Laboratory (Quebec, Canada), while MCH-tdTomato mice were bred at Memorial University and used to visualize MCH-expressing neurons. MCH-tdTomato mice were generated by crossing a *Mch-cre* mouse (originally generated by Dr. Brad Lowell, Harvard University and breeders were kindly provided by Dr. Melissa Chee, Carleton University) with a cre-dependent tdTomato reporter mouse (stock number 007909, Jackson Laboratory). All mice used were 6–12 weeks old at the time of experimentation. Animals were kept on a 12/12 h light/dark cycle and fed *ad libitum* with standard chow.

### Electrophysiology

Mice were deeply anesthetized with isoflurane and decapitated, and the brain was isolated. Coronal slices (250 μm) of the hypothalamus were obtained with the vibratome (VT-1000, Leica Microsystems) in cold artificial cerebrospinal fluid (ACSF) containing (mM): 126 NaCl, 2.5 KCl, 2 CaCl_2,_ 1.2 NaH_2_PO_4_, 1.2 MgCl_2_, 18 NaHCO_3_, 2.5 glucose, and bubbled with 95% O_2_/5% CO_2_. Slices were then incubated at 32°C in ACSF for 30 min or in recovery solution (in mM: 92 NMDG, 2.5 KCl, 1.25 NaH_2_PO_4_, 30 NaHCO_3_, 20 HEPES, 25 glucose, 5 ascorbic acid, 2 thiourea, 3 sodium pyruvate, 10 MgSO_4_, and 0.5 CaCl_2_) for 15 min before being transferred into ACSF for another 15 min. Then, slices were left in ACSF at room temperature until recording. Solutions were continuously bubbled with 95% O_2_/5% CO_2_. No differences in electrophysiological parameters were found in identified MCH neurons due to the recovery solution (data not shown), thus data were combined.

Hemisected brain slices were placed in the recording chamber and perfused continuously with ACSF at 27–30°C. An infrared differential interference contrast microscope (DM LFSA, Leica Microsystems) was used to visualize neurons. Whole cell patch clamping was performed using Multiclamp 700B and pClamp 10 software (Molecular Devices). An internal solution composed of (in mM): 123 K-gluconate, 2 MgCl_2_, 1 KCl, 0.2 EGTA, 10 HEPES, 5 Na_2_ATP, 0.3 NaGTP, and 2.7 biocytin was used to fill glass electrodes with a tip resistance of 3–6 MΩ. Once whole cell mode was attained, a series of hyperpolarizing and depolarizing current (600 ms) in 50-pA or 20-pA increments was applied in current clamp mode, and resulting voltage responses were filtered at 5 kHz and acquired at 10 kHz. To test the effect of tetrodotoxin (1 μM, Alomone Labs) or ZD 7288 (50 μM, Hello Bio), aliquots of the compounds were diluted to the final concentration in ACSF and applied in the bath.

### Immunohistochemistry

Following recording, immunohistochemistry was performed to confirm the neurochemical phenotype. First, brain slices were fixed in 10% neutral-buffered formalin overnight at 4°C following electrophysiology. Next, fixed slices were incubated in rabbit anti-MCH antibody (1:1000, H-070-47, Phoenix Pharmaceuticals Inc.) for 3 days at 4°C. Slices were then treated overnight with an appropriate secondary antibody and AMCA-streptavidin to visualize biocytin (1:500; 016–150-084, Jackson ImmunoResearch). For MCH-tdTomato mice, MCH peptide staining was not performed. To identify co-localization of CART in MCH neurons, brain slices were treated with a cocktail of goat anti-MCH antibody (1:500; sc14509, Santa Cruz Biotechnology Inc.) and rabbit anti-CART antibody (1:500; H-003-62, Phoenix Pharmaceuticals Inc.), followed by appropriate secondary antibodies and AMCA-streptavidin. Stained sections were imaged using a confocal or epifluorescence microscope to identify the co-localization of biocytin with MCH or tdTomato and CART. Only cells that were confirmed to be MCH neurons (i.e., MCH-immunoreactive or tdTomato-positive) were included in the analysis.

### Data analysis

Membrane capacitance (Cm) and resistance (Rm) were measured in voltage clamp mode by applying 5-mV step pulses at a holding potential of −70 mV using the membrane test function of pClamp. Membrane time constant (*τ*) was calculated as τ = Cm × Rm. Unique electrophysiological responses to hyperpolarizing and depolarizing current injections were used to identify putative MCH neurons (Parsons and Hirasawa, 2011; Linehan et al., 2015) and to assess active and passive membrane properties using Clampfit 10 (Molecular devices) as previously described (Linehan and Hirasawa, 2018). Briefly, the number of action potentials (APs) and the latency to first spike were assessed during 600-ms positive current injections. AP waveform was analyzed using the following definitions: AP threshold was the membrane potential where the slope of the trace reached 10 mV/ms; AP amplitude was the membrane potential from the baseline to the peak, where baseline was defined as the membrane potential 30 ms before the threshold; half-width was the time between the rise and decay phase of AP at half amplitude; and after hyperpolarization (AHP) amplitude was the difference between baseline and the peak of AHP. If no AP was elicited, the duration of the current injection (600 ms) was assigned as the latency and AP waveform analysis was not performed. In case an AP was fired immediately after the start of positive current injection, the second AP was analyzed; otherwise, the first AP was used for AP waveform analysis. Voltage sag/H-current amplitude was the difference between the peak of membrane hyperpolarization and the steady-state membrane potential at the end of hyperpolarizing current injections. H-current density was calculated by dividing H-current amplitude by Cm. All recorded membrane potentials were corrected for liquid junction potential (−14.9 mV according to pClamp LJP calculator). Cells with series/access resistance greater than 25 MΩ were excluded from analysis.

Statistical tests were performed using Prism 9 (GraphPad Software Inc), *p* < 0.05 was considered significant. Two-way ANOVA (ordinary or repeated measures) was performed to compare multiple groups in both sexes and when significant main effect or interaction was found, Tukey’s multiple comparison test was performed. One-way ANOVA was used when male and female cells were pooled. Simple linear regression analysis was performed to assess for any correlation between two factors. Kruskal–Wallis test was used for assessing H-currents in different cell subgroups. The sample size and main effects are indicated in Table 1 and figure legend, while the post-test results are shown in the figures. Results are presented as mean ± SEM. Quantile–quantile (Q–Q) plots of indicated variables were generated in Prism 9 and are shown in Supplementary Figure S1.

**Table 1:** Sample size and statistical test information. *p*-values that reached significance (i.e. *p*<0.05) are bolded.

Figure panel	Sample size cells (n)/animals (N)	Test(s)	*p*-value, *R*^2^	*F*, *t*
Figure 2				
2B Cm	Male ZI = 27/19 Female ZI = 10/7 Male LH = 22/12 Female LH = 17/7 Male MH = 23/17 Female MH = 14/9	2 way ANOVA	Interaction: 0.3659 Area: **0.0006** Sex: 0.6498	Interaction: *F*(2,107) = 1.015 Area: *F*(2,107) = 7.931 Sex: *F*(1,107) = 0.2073
2C Rm	Male ZI = 26/18; Female ZI = 10/7 Male LH = 22/12 Female LH = 17/7 Male MH = 23/17 Female MH = 14/9	2 way ANOVA	Interaction: 0.3453 Area: 0.0733 Sex: 0.8782	Interaction: *F*(2,106) = 1.074 Area: *F*(2,106) = 2.678 Sex: *F*(1,106) = 0.02360
2D Time const	Male ZI = 27/19 Female ZI = 10/7 Male LH = 22/12 Female LH = 17/7 Male MH = 23/17 Female MH = 14/9	2 way ANOVA	Interaction: 0.3876 Area: **0.0001** Sex: 0.7912	Interaction: *F*(2,107) = 0.9562 Area: *F*(2,107) = 10.04 Sex: *F*(1,107) = 0.07042
2E RMP		2 way ANOVA	Interaction: 0.8973 Area: **0.0025** Sex: 0.6472	Interaction: *F*(2,107) = 0.1085 Area: *F*(2,107) = 6.344 Sex: *F*(1,107) = 0.2106
2F	Male ZI = 27/19 Female ZI = 10/7	Simple linear regression	Male ZI: *p* = 0.8143. R^2^ = 0.002248 Female ZI: *p* = 0.3884. *R*^2^ = 0.09417 Male ZI vs. Female ZI Slopes *p* = 0.4225	Male: *F*(1,25) = 0.05632 Female: *F*(1,8) = 0.8317 Slopes *F*(1,33) = 0.6598
2G			Male ZI: *p* = **0.0147**. *R*^2^ = 0.2237 Female ZI: *p* = 0.2338. *R*^2^ = 0.1717 Male ZI vs. Female ZI slopes *p* = 0.9235	Male ZI: *F*(1,24) = 6.917 Female ZI: *F*(1,8) = 1.658 Slopes *F*(1,32) = 0.009361
2H			Male ZI: *p* = **0.0021**. *R*^2^ = 0.3192 Female ZI: *p* = 0.0839. *R*^2^ = 0.3275 Male ZI vs. Female ZI slopes *p* = 0.9039	Male ZI: *F*(1,25) = 11.72 Female ZI: *F*(1,8) = 3.896 Slopes *F*(1,33) = 0.01480
2I	Male LH = 22/12 Female LH = 17/7	Simple linear regression	Male LH: *p* = **<0.0001**. *R*^2^ = 0.5676 Female LH: *p* = 0.**0089**. *R*^2^ = 0.3757 Male LH vs. Female LH *p* = 0.7107	Male LH: *F*(1,20) = 26.25 Female LH: *F*(1,15) = 9.026 Slopes *F*(1,35) = 0.1398
2J			Male LH: *p* = **0.0088**. *R*^2^ = 0.2965 Female LH: *p* = 0.7819. *R*^2^ = 0.005270 Male LH vs. Female LH *p* = 0.0825	Male LH: *F*(1,20) = 8.430 Female LH: *F*(1,15) = 0.07946 Slopes *F*(1,35) = 3.196
2K			Male LH: *p* = 0.3192. *R*^2^ = 0.04959 Female LH: *p* = 0.0767. *R*^2^ = 0.1941 Male LH vs. Female LH *p* = 0.6499	Male LH: *F*(1,20) = 1.044 Female LH: *F*(1,15) = 3.614 Slopes *F*(1,35) = 0.2096
2L	Male MH = 23/17 Female MH = 14/9	Simple linear regression	Male MH: *p* = 0.9651. *R*^2^ = 9.328e-005 Female MH: *p* = 0.1818. *R*^2^ = 0.1434 Male MH vs. Female MH *p* = 0.3163	Male MH: *F*(1,21) = 0.001959 Female MH: *F*(1,12) = 2.009 Slopes *F*(1,33) = 1.036
2M			Male MH: *p* = 0.6313. *R*^2^ = 0.01117 Female MH: *p* = 0.9456. *R*^2^ = 0.0004050 Male MH vs. Female MH *p* = 0.8032	Male MH: *F*(1,21) = 0.2371 Female MH: *F*(1,12) = 0.004863 Slopes *F*(1,33) = 0.06314
2N			Male MH: *p* = 0.6993. *R*^2^ = 0.007248 Female MH: *p* = 0.2909. *R*^2^ = 0.09233 MH vs. Female MH *p* = 0.7175	Male MH: *F*(1,21) = 0.1533 Female MH: *F*(1,12) = 1.221 Slopes *F*(1,33) = 0.1332
Figure 3				
3A Latency	Male ZI = 27/19 Female ZI = 10/7 Male LH = 22/12 Female LH = 17/7 Male MH = 23/17 Female MH = 14/9	2 way ANOVA	Interaction: 0.3383 Area: **0.0122** Sex: 0.8042	Interaction: *F*(2,105) = 1.095 Area: *F*(2,105) = 4.595 Sex: *F*(1,105) = 0.06178
3B AP freq		2 way ANOVA	Interaction: **0.0025** Area: **0.0001** Sex: 0.1972	Interaction: *F*(2,105) = 6.350 Area: *F*(2,105) = 12.21 Sex: *F*(1,105) = 1.684
3C Threshold	Male ZI = 6/5 Female ZI = 3/3 Male LH = 17/9 Female LH = 7/5 Male MH = 13/11 Female MH = 10/6	2 way ANOVA	Interaction: 0.1532 Area: 0.4156 Sex: 0.4449	Interaction: *F*(2,50) = 1.948 Area: *F*(2,50) = 0.8937 Sex: *F*(1,50) = 0.5931
3D AP amp		2 way ANOVA	Interaction: 0.6724 Area: 0.1654 Sex: 0.5805	Interaction: *F*(2,50) = 0.4001 Area: *F*(2,50) = 1.866 Sex: *F*(1,50) = 0.3095
3E AHP amp		2 way ANOVA	Interaction: 0.1415 Area: 0.2845 Sex: 0.2127	Interaction: *F*(2,50) = 2.034 Area: *F*(2,50) = 1.289 Sex: *F*(1,50) = 1.594
3F Half width		2 way ANOVA	Interaction: 0.5851 Area: **0.0279** Sex: 0.0808	Interaction: *F*(2,50) = 0.5418 Area: *F*(2,50) = 3.846 Sex: *F*(1,50) = 3.176
Figure 4				
4B AP freq	ZI = 8/7 LH =11/7 MH = 9/8	2 way ANOVA	Interaction: **0.0006** Driving current: **<0.0001** Area: 0.0757	Interaction: *F*(6,72) = 4.491 Driving current: *F*(3,72) = 100.5 Area: *F*(2,24) = 2.880
4C Latency		2 way ANOVA	Interaction: **0.0385** Driving current: **<0.0001** Area: 0.1384	Interaction: *F*(6,72) = 2.364 Driving current: *F*(3,72) = 56.92 Area: *F*(2,24) = 2.150; 0.1384
4D Threshold		1-way ANOVA	**0.0065**	*F*(2,25) = 6.197
4E AP amp		1-way ANOVA	**0.0487**	*F*(2,25) = 3.420
4F half width		1-way ANOVA	**0.0187**	*F*(2,24) = 4.719
4G AHP amp		1-way ANOVA	**0.0211**	*F*(2,24) = 4.548
Figure 6				
6B Cm	Males LH CART− = 15/8 LH CART+ = 4/3 ZI CART+ = 20/13 MH CART+ = 12/8 Females LH CART− = 9/4 LH CART+ = 8/3 ZI CART+ = 9/7 MH CART+ = 14/8	2 way ANOVA	Interaction: 0.3273 Area: **0.0002** Sex: 0.5952	Interaction: *F*(3,83) = 1.167 Area: *F*(3,83) = 7.179 Sex: *F*(1,83) = 0.2844
6C Rm		2 way ANOVA	Interaction: 0.5018 Area: 0.0721 Sex: 0.5006	Interaction: *F*(3,83) = 0.7920 Area: *F*(3,83) = 2.417 Sex: *F*(1,83) = 0.4576
6D Tau		2 way ANOVA	Interaction: 0.2609 Area: **0.0009** Sex: 0.3840	Interaction: *F*(3,83) = 1.360 Area: *F*(3,83) = 6.056 Sex: *F*(1,83) = 0.7658
6E RMP		2 way ANOVA	Interaction: 0.3693 Area: **<0.0001** Sex: 0.7731	Interaction: *F*(3,83) = 1.063 Area: *F*(3,83) = 9.134; Sex: *F*(1,83) = 0.08370
6F AP freq	Males LH CART− = 15/8 LH CART+ = 4/3 ZI CART+ = 20/13 MH CART+ = 12/8 Females LH CART− = 8/4 LH CART+ = 8/3 ZI CART+ = 9/7 MH CART+ = 14/8	2 way ANOVA	Interaction: 0.3144 Area: **<0.0001** Sex: 0.5726	Interaction: *F*(3,82) = 1.202 Area: *F*(3,82) = 15.00 Sex: *F*(1,82) = 0.3210
6G Latency		2 way ANOVA	Interaction: 0.4103 Area: **<0.0001** Sex: 0.3908	Interaction: *F*(3,82) = 0.9715 Area: *F*(3,82) = 11.00 Sex: *F*(1,82) = 0.7444
6H Adaptation	CART−M = 12/7 CART−F = 6/4 CART+M = 9/9 CART+F = 8/6	2 way ANOVA	Interaction: 0.3382 CART: **0.0101** Sex: 0.1169	Interaction: *F*(1,30) = 0.9474 CART: *F*(1,30) = 7.538 Sex: *F*(1,30) = 2.606
6I Threshold	CART−M = 12/7 CART−F = 6/4 CART+M = 13/12 CART+F = 14/10	2 way ANOVA	Interaction: 0.1708 CART: 0.6658 Sex: 0.6345	Interaction: *F*(1,41) = 1.943 CART: *F*(1,41) = 0.1893 Sex: *F*(1,41) = 0.2295
6J AP amp		2 way ANOVA	Interaction: 0.7551 CART: 0.5465 Sex: 0.2761	Interaction: *F*(1,41) = 0.09861 CART: *F*(1,41) = 0.3697 Sex: *F*(1,41) = 1.218
6K AHP amp		2 way ANOVA	Interaction: 0.2342 CART: 0.4778 Sex: 0.8184	Interaction: *F*(1,41) = 1.458 CART: *F*(1,41) = 0.5132 Sex: *F*(1,41) = 0.05338
6L Half width		2 way ANOVA	Interaction: 0.7601 CART: **0.0128** Sex: **0.0490**	Interaction: *F*(1,41) = 0.09445 CART: *F*(1,41) = 6.781 Sex: *F*(1,41) = 4.118
Figure 7				
7A	Males LH CART− = 14/8 LH CART+ = 4/3 ZI CART+ = 19/13 MH CART+ = 12/8	Kruskal–Wallis test	***p* < 0.0001**	
7B	Females LH CART− = 7/4 LH CART+ = 8/3 ZI CART+ = 9/7 MH CART+ = 14/9	Kruskal–Wallis test	***p* < 0.0001**	
7C	Males LH CART− = 14/8 LH CART+ = 4/3 ZI CART+ = 19/13 MH CART+ = 12/8	2 way ANOVA	Interaction: <**0.0001** Current: **0.0008** Area: **<0.0001**	Interaction: *F*(9,135) = 8.352 Current: *F*(3,135) = 5.948 Area: *F*(3,45) = 13.12
7D	Females LH CART− = 7/4 LH CART+ = 8/3 ZI CART+ = 9/7 MH CART+ = 14/9	2 way ANOVA	Interaction: **0.0081** Current: **0.0203** Area: **<0.0001**	Interaction: *F*(9,136) = 2.614 Current: *F*(3,136) = 3.375 Area: *F*(3,136) = 15.89
7E	Males LH CART− = 14/8 LH CART+ = 4/3 ZI CART+ = 19/13 MH CART+ = 12/8 Females LH CART− = 7/4 LH CART+ = 8/3 ZI CART+ = 9/7 MH CART+ = 14/9	2 way ANOVA	Interaction: 0.9288 Current: **<0.0001** Area: 0.7350	Interaction: *F*(3,79) = 0.1509 Current: *F*(3,79) = 15.68 Area: *F*(1,79) = 0.1153
7G	CART− 5/3	2 way RM ANOVA	Interaction: 0.0886 Treatment: **0.0177** Current: 0.0886	Interaction: *F*(9,36) = 1.871 Treatment: *F*(1,4) = 15.14 Current: *F*(9,36) = 1.871
7H		Linear regression	Baseline: *p* **< 0.0001**, *R*^2^ = 0.9809 + ZD7288: *p* **< 0.0001,** *R*^2^ = 0.9974 Slopes **<0.0001**	Baseline: *F*(1,63) = 3,238; +ZD7288: *F*(1,62) = 23,916 Slopes *F* (1,125) = 35.27
7I	CART+ = 4/3	2 way RM ANOVA	Interaction: 0.5941 Treatment: 0.2500 Current: 0.5317	Interaction: *F*(9,27) = 0.8310 Treatment: *F*(1,3) = 2.024 Current: *F*(9,27) = 0.9091
7K	CART− = 21/12 CART− depol = 7/7 CART− hyperpol = 54/22	1 way ANOVA	**<0.0001**	*F*(2,79) = 27.24
7L	Males LH CART− = 14/8 LH CART+ = 4/3 ZI CART+ = 19/13 MH CART+ = 12/8	2 way ANOVA	Interaction: **<0.0001** Current: **<0.0001** Area: **<0.0001**	Interaction: *F*(12,180) = 21.01 Current: *F*(4,180) = 209.3 Area: *F*(3,45) = 10.83
7M	Females LH CART− = 7/4 LH CART+ = 8/3 ZI CART+ = 9/7 MH CART+ = 14/9	2 way ANOVA	Interaction: **<0.0001** Current: **<0.0001** Area: **0.0368**	Interaction: *F*(12,136) = 15.90 Current: *F*(1.265,43.02) = 282.9 Area: *F*(3,34) = 3.166

## Results

**Figure 1:**
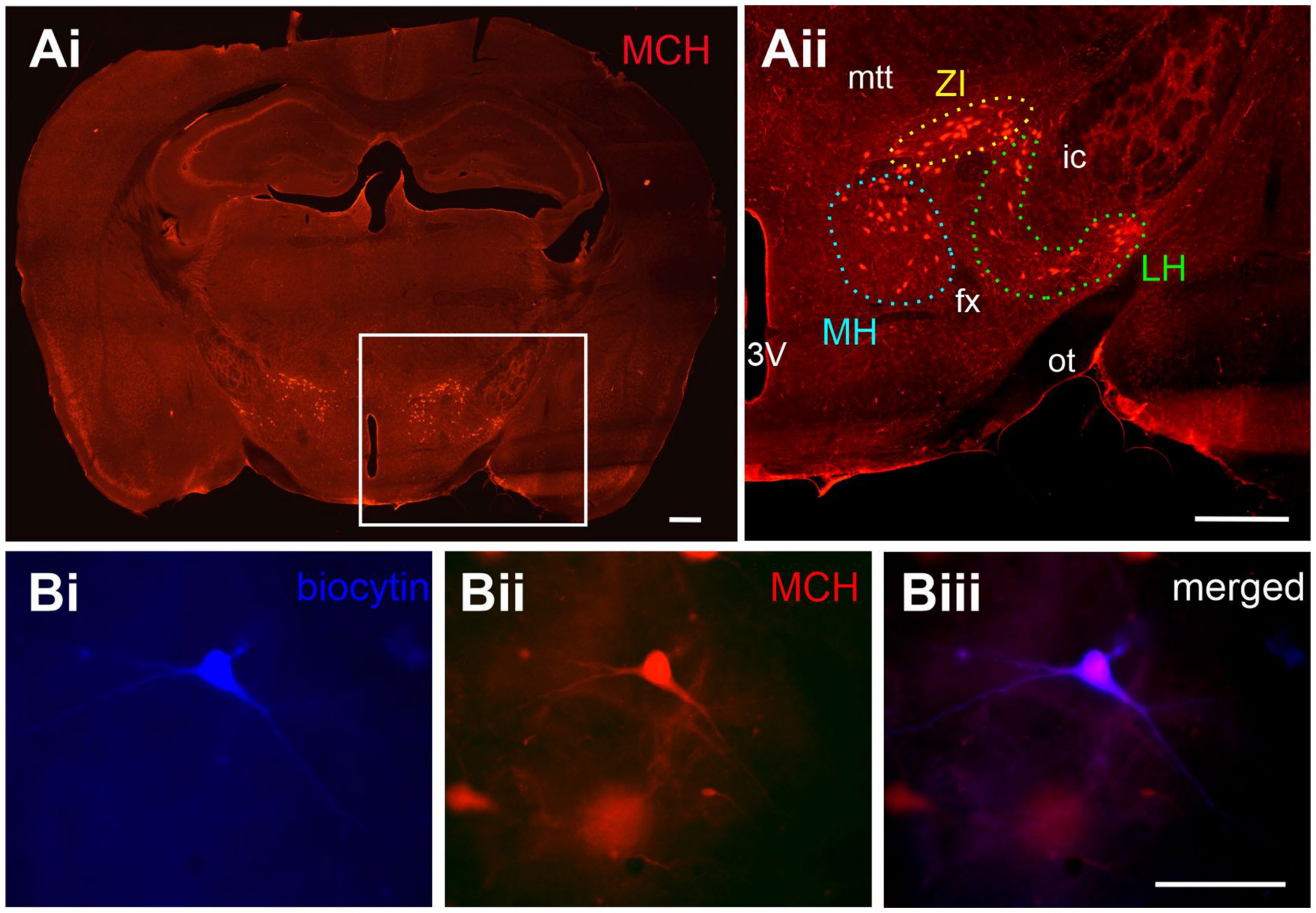
Anatomical distribution of MCH neurons. **(A)** MCH immunoreactivity in the mouse brain. An area with a high density of MCH neurons in the whole coronal brain image (**Ai**, white box) is shown at a higher magnification **(Aii)**. Anatomical subpopulations of MCH neurons were identified within the medial hypothalamus (MH), lateral hypothalamus (LH) and zona incerta (ZI) according to their position relative to the third ventricle (3V), fornix (fx), optic tract (ot), mammillothalamic tract (mtt) and internal capsule (ic). **(B)** Representative image of a neuron filled with biocytin during patch clamp recording **(Bi)** and later confirmed to co-localize with the MCH peptide **(Bii,Biii)**. Scale bar: **(Ai,Aii)** 500 μm. **(Bi-Biii)** 100 μm.

**Figure 2:**
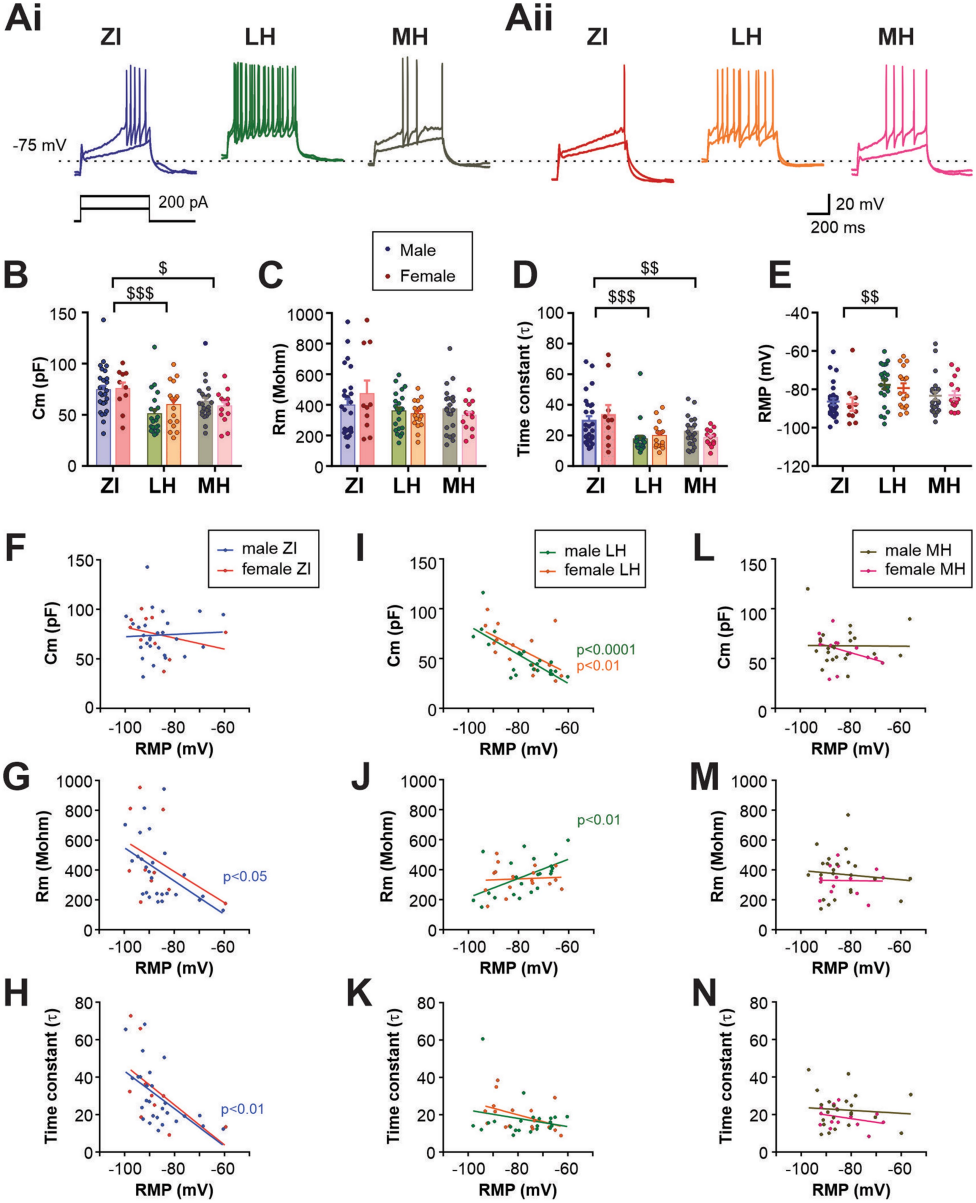
Electrophysiological characteristics of anatomical subpopulations of MCH neurons in male and female mice. **(Ai,Aii)** Representative *ex-vivo* current clamp recording of MCH neurons from male **(Ai)** and female mice **(Aii)** in three anatomical regions as indicated. Traces show the response to 100- and 200-pA driving currents for 600 ms. **(B–D)** Membrane capacitance (Cm, **B**), resistance (Rm, **C**) and time constant (*τ*, **D**) of MCH subpopulations measured at −70 mV in voltage clamp mode. **(E)** Resting membrane potential (RMP) of MCH neurons in different anatomical regions. **(F–N)** Correlation analysis between RMP and other passive membrane properties of MCH neurons. Male and female MCH neurons are grouped according to their anatomical localization in the ZI **(F,G)**, LH **(I–K),** and **(L–N)**. Data shown in panels **B–E** are used for the regression analysis. ^$^*p* < 0.05, ^$$^*p* < 0.01, ^$$$^*p* < 0.001 multiple comparisons between areas. Mean ± SEM are shown.

Melanin-concentrating hormone neurons, visualized by immunohistochemistry or tdTomato expression, were distributed in loose clusters in three anatomical areas (Figure 1A), namely the ZI, LH (ventral to the ZI and lateral to the fornix), and the MH (medial to the fornix). We performed patch clamp recordings of MCH neurons (Figure 1B) and classified them into subgroups according to their localization in these three anatomical areas for comparison of their electrophysiological properties (Figure 2A).

Among the passive membrane properties examined, no effect of sex was observed (main effect and interaction), while area differences were found in membrane capacitance (Cm) and time constant (*τ*) but not in membrane resistance (Rm) (Figures 2B–D). ZI cells had a greater Cm, which corresponded with a larger soma area of biocytin-filled cells in the ZI (Supplementary Figure S2), suggesting that the difference in soma size accounts for the larger Cm. Area-dependent differences were also seen in the resting membrane potential (RMP, Figure 2E). The application of a voltage-gated Na^+^ channel blocker tetrodotoxin did not alter RMP, thus this area difference is due to intrinsic properties (Supplementary Figure S3). As the RMP values were highly variable within each group, regression analysis between RMP and other passive membrane properties was performed (Figures 2F–N). We found that in male ZI cells, RMP was negatively correlated with Rm or time constant (Figures 2G,H), suggesting that hyperpolarized ZI cells may have greater membrane permeability at rest. RMP of LH cells was negatively correlated with Cm in both males and females (Figure 2I), whereas RMP and Rm were positively correlated in male LH cells (Figure 2J). Thus, depolarized cells in the LH may be smaller and have higher membrane resistance. No significant correlation between RMP and other measures were found in the MH.

**Figure 3:**
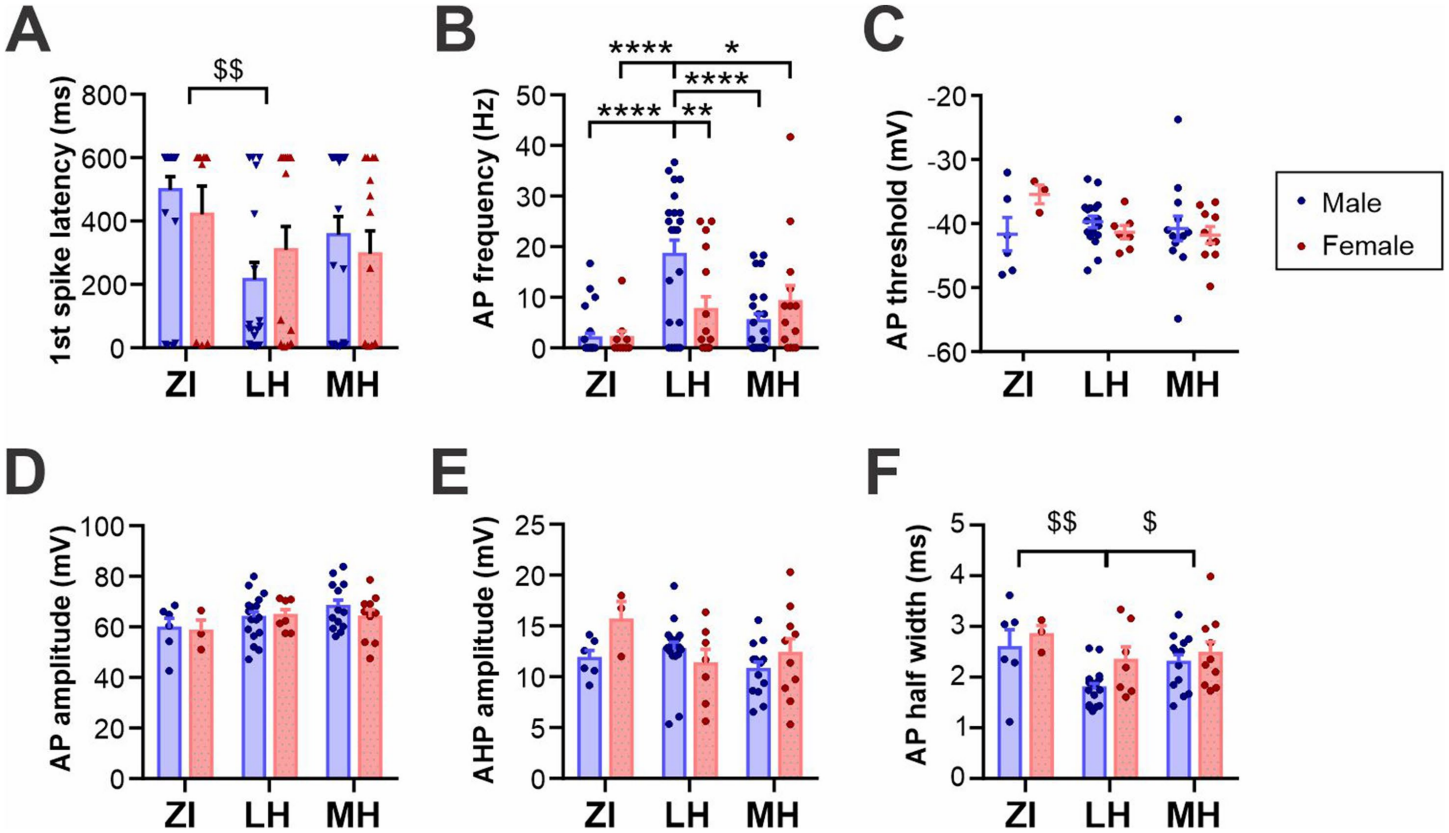
Active membrane properties of anatomical subpopulations of MCH neurons. **(A,B)** Latency to the first action potential (AP) **(F)** and AP frequency **(G)** during 600-ms, 200 pA driving current injections. When no AP was elicited, the latency was noted as 600 ms. **(C,D)** AP waveform analysis of MCH subpopulations showing the AP threshold **(H)** and amplitude **(I)**. **(E)** Amplitude of afterhyperpolarization (AHP). **(F)** Half-width of APs. *p < 0.05, **p < 0.01, ****p < 0.0001 multiple comparisons between individual groups. ^$^*p* < 0.05, ^$$^*p* < 0.01 multiple comparisons between areas. Mean ± SEM are shown.

When stimulated by positive driving current injections, the latency and frequency of elicited AP were also found to be different among the areas (Figures 3A,B and Supplementary Figure S4). Specifically, ZI MCH neurons were the least excitable with hyperpolarized RMP and less evoked firing, while LH MCH neurons were the most excitable, particularly those of male mice. The AP waveform was analyzed for cells that fired APs during positive driving currents, which revealed that AP threshold, AP amplitude and AHP amplitude were similar among spatially-defined groups and between sexes (Figures 3C–E). The AP half-width, however, was shorter in LH cells without differences between the sexes (Figure 3F).

**Figure 4:**
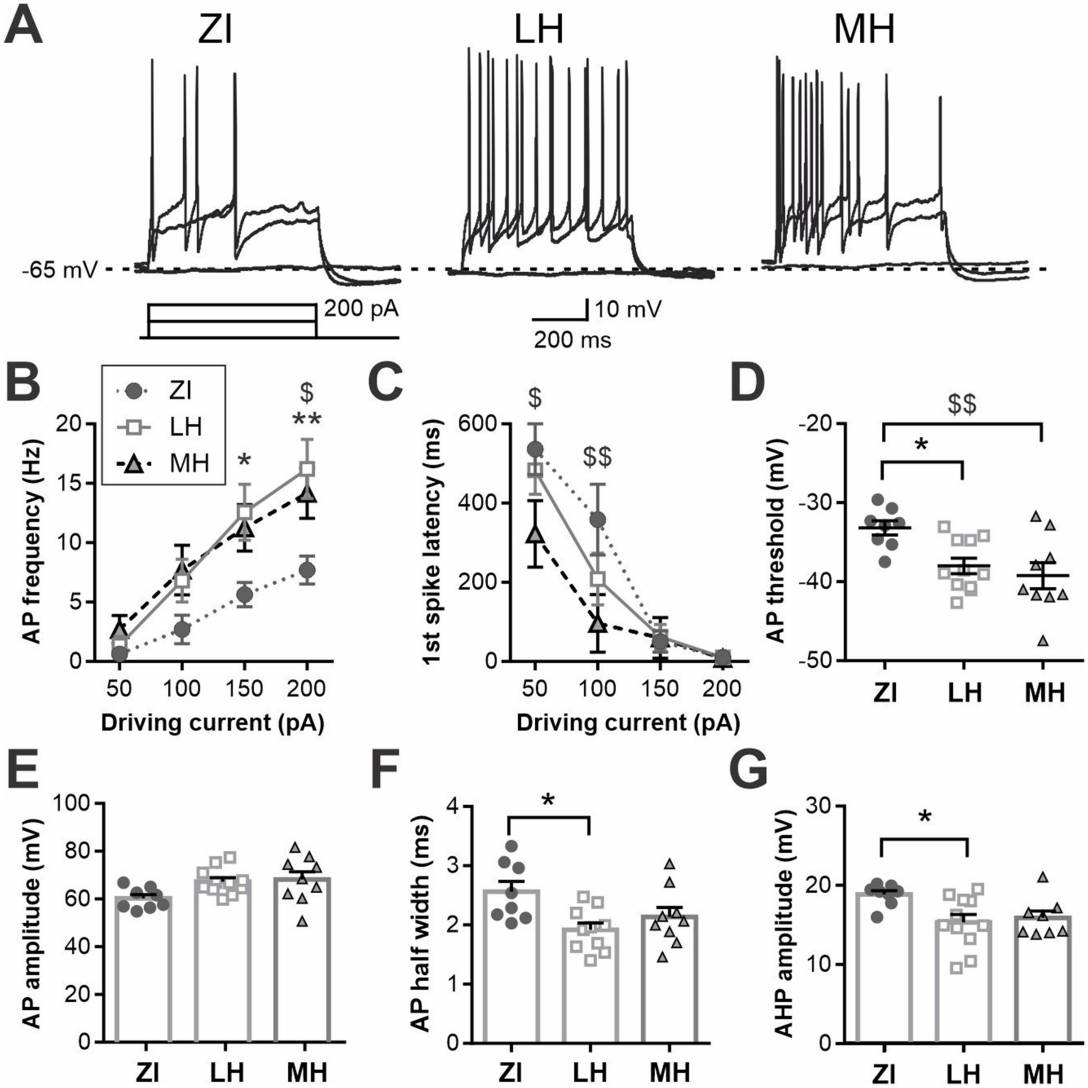
Differential excitability of anatomical subpopulations of MCH neurons is not solely due to the difference in RMP. **(A)** Representative recording of MCH neurons at a subthreshold potential (−65 mV) and challenged with driving currents. **(B,C)** AP frequency **(B)** and latency to 1st spike **(C)** during driving current injections. **(D–G)** AP waveform measures showing threshold **(D)**, amplitude **(E)**, half-width **(F)** of APs and AHP amplitude **(G)**. *p < 0.05, **p < 0.01 LH vs. ZI; ^$^*p* < 0.05, ^$$^*p* < 0.01 ZI vs. MH. Mean ± SEM are shown.

We noted that approximately half of MCH neurons recorded remained silent when challenged with positive current injections, even during the maximum driving current used in this study (+200 pA). Thus, to further assess the electrophysiological properties of MCH neurons, a subset of these neurons was held at a subthreshold potential (−65 mV) and then positive driving currents were applied (Figure 4). As no difference in RMP was found between male and females in the three anatomical regions, cells from both sexes were combined for this assessment. We found that MCH neurons within different brain areas had distinct levels of excitability, as measured by AP frequency (Figure 4B) and the first spike latency (Figure 4C). Specifically, ZI-MCH neurons were less excitable than those in other areas despite being held at the same baseline holding potential. An AP waveform analysis of these recordings revealed area-dependent differences in AP threshold, half-width and AHP amplitude, but not AP amplitude (Figures 4D–G).

**Figure 5:**
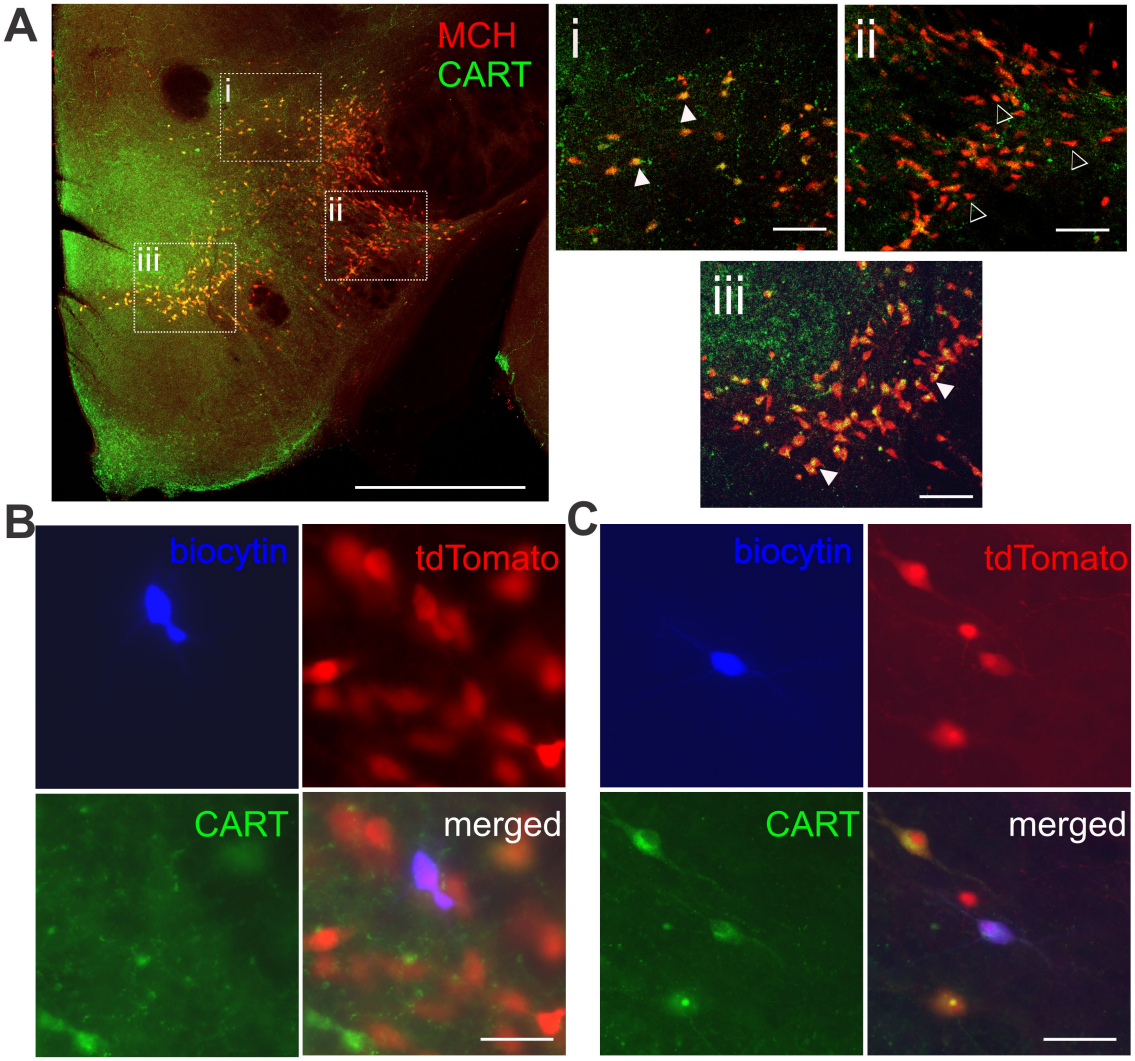
Immunohistochemical detection of CART co-expression in MCH neurons. **(A)** Double immunofluorescence of MCH and CART. Boxed areas in the left image show the ZI **(i)**, the LH **(ii)**, and the MH **(iii)**, enlarged in the insets (right). MCH neurons with co-localization of CART (filled arrowheads) are more common in the ZI and MH. MCH/CART− neurons (open arrowheads) are more frequently found in the LH. Scale bar: (Left) 1 mm, (Right, insets) 100 μm. **(B,C)** Representative images of tdTomato-expressing MCH neurons filled with biocytin during patch clamp recording, classified as CART-negative **(B)** or CART-positive **(C)**. Scale bar: 50 μm.

**Figure 6:**
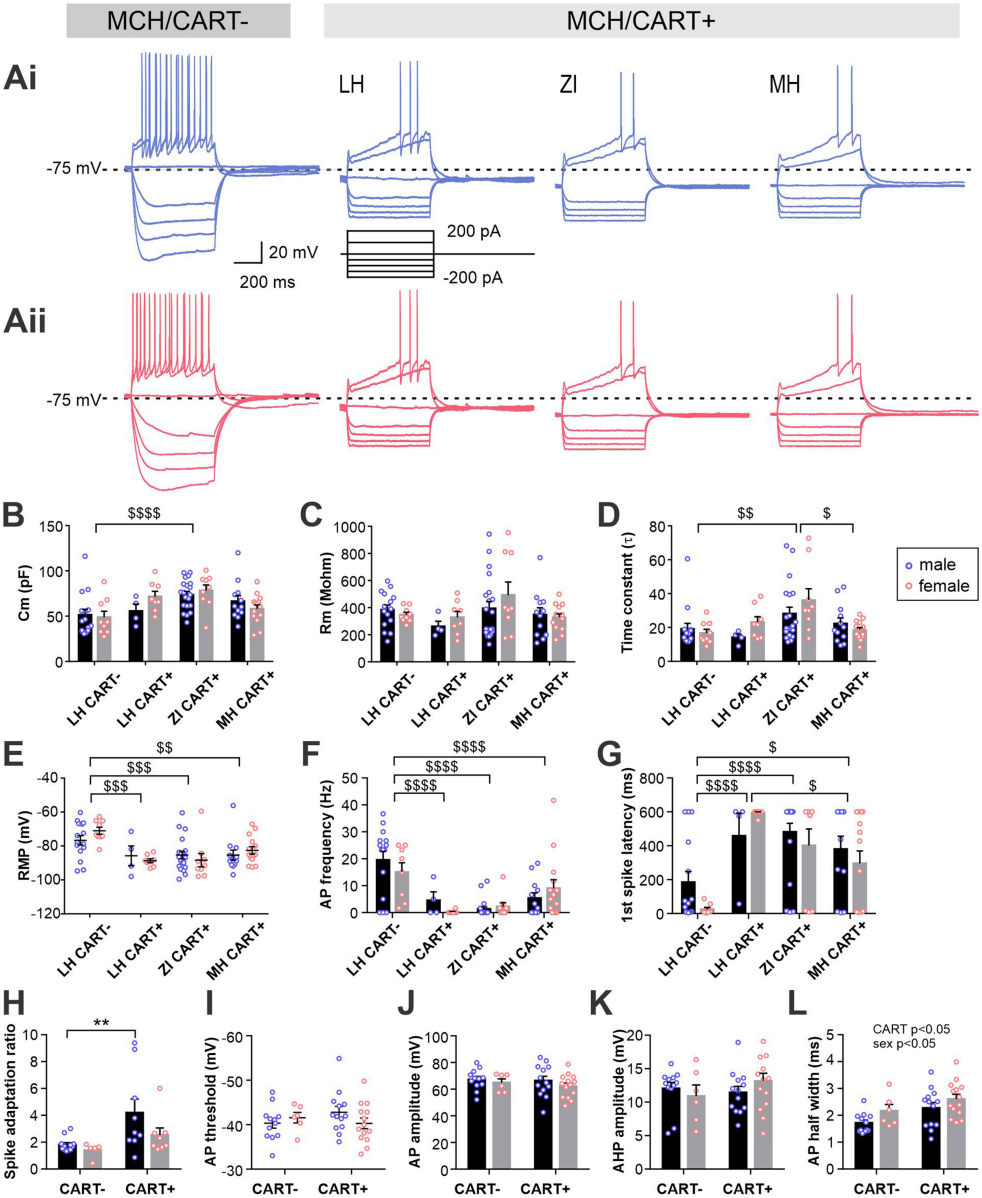
Electrophysiological characteristics of MCH neurons with or without CART expression. **(Ai,Aii)** Representative current clamp recording of CART-negative and -positive MCH neurons in different brain regions in male **(Ai)** and female mice **(Aii)**. **(B–E)** MCH neurons are grouped according to the CART expression and anatomical localization: MCH/CART− cells in LH (LH CART−) and MCH/CART+ cells in LH, ZI and MH. Graphs show passive membrane properties including Cm **(B)**, Rm **(C)**, time constant **(D)** and RMP **(E)**. **(F,G)** AP frequency **(F)** and latency to first AP **(G)** during 600-ms, 200-pA current injections. **(H)** Spike adaptation (ratio of first and last AP intervals). **(I–L)** Comparisons of AP waveform characteristics between male and female MCH neurons with or without CART expression, including AP threshold **(I)**, AP amplitude **(J)**, AHP amplitude **(K)** and half-width **(L)**. MCH/CART+ cells in all three anatomical areas are combined. ^$^*p* < 0.05, ^$$^*p* < 0.01, ^$$$^*p* < 0.001, ^$$$$^*p* < 0.0001, multiple comparisons between areas. **p < 0.01, multiple comparison between individual groups. Mean ± SEM are shown.

Another way to classify MCH neurons is by the co-expression of CART. CART expression was found in regions with a high concentration of MCH neurons (Figure 5A). Most CART immunopositive (CART+) MCH neurons were found localized to the ZI and MH, while double staining was far less common in the LH. Consequently, we were only able to conduct electrophysiological assessment on MCH/CART− cells within the LH, which were then compared to MCH/CART+ cells in all three regions (Figures 5B,C, 6A).

This comparison of MCH neuron subgroups revealed a significant difference in Cm and time constant without any difference in Rm (Figures 6B–D), corroborating our initial finding that ZI CART+ cells were larger (i.e., larger membrane area). There was a striking difference in RMP, where LH CART− cells had a more depolarized RMP than CART+ cells in all three regions (Figure 6E). Furthermore, when APs were evoked by depolarizing currents, LH CART− cells had a shorter firing latency and fired more frequently than CART+ cells (Figures 6F,G and Supplementary Figure S5).

For additional analysis of APs, data from different anatomical regions were combined, as MCH/CART+ cells were less excitable and often did not fire any APs. Among the cells that fired at least four APs, which allowed the assessment of the spike adaptation ratio, CART+ cells were found to show greater adaptation than CART− cells, particularly in males (Figure 6H). A comparison of the AP waveform found no differences in AP threshold, AP amplitude or AHP amplitude according to the neurochemical identity or sex (Figures 6I–K). However, a main effect of CART expression and sex were found for the AP half-width (Figure 6L). Taken together, these results indicate that MCH/CART− cells in LH are more excitable than MCH/CART+ cells in LH, ZI and MH, whereas MCH/CART+ cell have similar electrophysiological properties regardless of their location.

**Figure 7:**
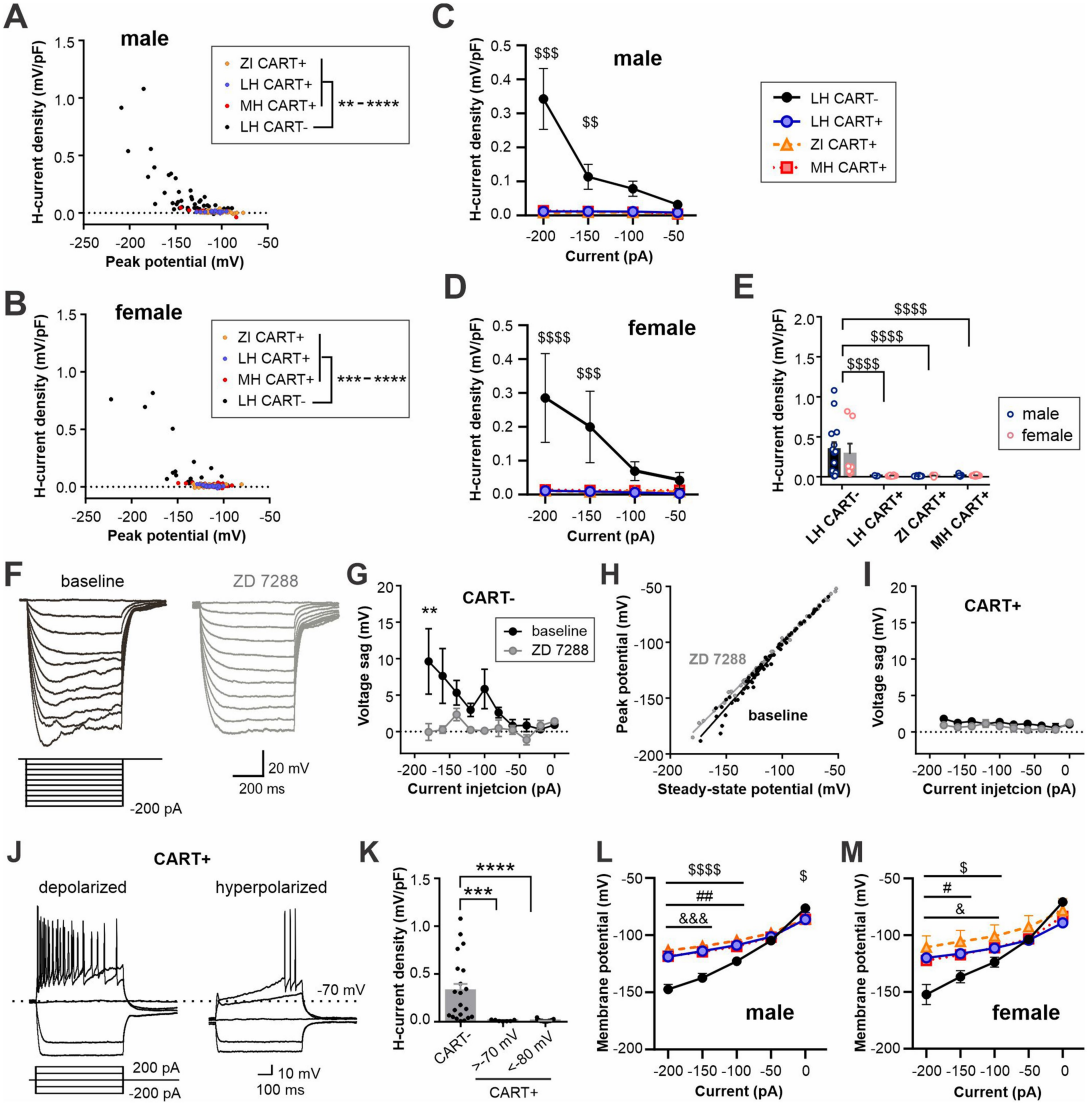
CART-negative MCH neurons display H-currents. **(A,B)** X–Y scatter plot of H-current density recorded after the peak membrane potential reached in response to hyperpolarizing current steps applied to male **(A)** and female **(B)** MCH neurons. The current steps ranged from −200 to −50 pA. Values from individual cells are pooled according to cell type. **(C,D)** H-current density in response to a range of hyperpolarizing current injections in male **(C)** and female cells **(D)**. **(E)** A summary plot showing H-current density recorded from −200 pA injection. **(F)** Representative current clamp recording from a MCH/CART− cell before (baseline) and during ZD 7288 application. **(G)** Repeated measures of voltage sag at baseline and in the presence of ZD 7288 in MCH/CART− cells. **(H)** X–Y scatter plot of the membrane potential at the peak of a voltage sag and ensuing steady-state in MCH/CART− cells shown in **(G)**. **(I)** Repeated measures of voltage sag at baseline and in the presence of ZD 7288 in MCH/CART+ cells. **(J)** Example recording of depolarized and hyperpolarized CART-positive MCH neurons. A minor population of MCH/CART+ neurons shows depolarized RMP like that of CART− neurons (>−70 mV) but lacks H-current. **(K)** A summary graph showing that H-current is unique to MCH/CART− neurons. **(L,M)** Steady-state potential in response to 600-ms hyperpolarizing current steps of varying magnitude in male **(L)** and female **(M)** MCH neurons. **(C–E)** ^$$^*p* < 0.01, ^$$$^*p* < 0.001, ^$$$$^*p* < 0.0001, LH CART− vs. all CART+ groups. **(G,K)** **p < 0.01, ***p < 0.001, ****p < 0.0001. **(L,M)** ^$^*p* < 0.05, ^$$$$^*p* < 0.0001, LH CART− vs. ZI; ^#^*p* < 0.05, ^##^*p* < 0.01, LH CART− vs. LH CART+; ^&^*p* < 0.05, ^&&&^*p* < 0.001, LH CART− vs. MH. Mean ± SEM are shown. For **(G–K)**, male and female cells were pooled since no sex differences were found in H-current density **(E)**.

One of the hallmarks of MCH neurons is the lack of an H-like current (Fang et al., 2023; Belanger-Willoughby et al., 2016). Surprisingly, however, some MCH neurons displayed a voltage sag, defined as the difference between the peak membrane potential and subsequent stead-state potential reached upon injection of hyperpolarizing currents, which was specific to CART− neurons in both males and females. MCH/CART+ neurons did not show any voltage sag regardless of anatomical region (Figures 7A–E). This voltage sag was inhibited by the H-current inhibitor ZD 7288 (Figures 7F–H).

Since MCH/CART− neurons had relatively depolarized RMP, it is possible that this is a characteristic of depolarized MCH neurons regardless of CART expression. However, we found that MCH/CART+ neurons, either depolarized (RMP > −70 mV), like CART− cells, or hyperpolarized (RMP < −80 mV), did not show a significant H-current (Figures 7J,K). Thus, H-current is a unique feature of MCH/CART− neurons, which has previously gone unnoticed.

Another unique feature of MCH neurons is inward rectification at negative membrane potentials. The steady-state membrane potential reached during a series of current injections displayed inward rectification in MCH/CART+ cells, which was more prominent than that in MCH/CART− cells in both males and females (Figures 7L,M), in agreement with a previous study (Miller et al., 2024).

## Discussion

The present study shows that discrete subpopulations of MCH neurons have distinct electrophysiological characteristics. Our primary finding denotes MCH neurons found in the LH to be more excitable than those found in the ZI or MH. When these cells were held at the same subthreshold potential, this difference in excitability between LH and ZI cells persisted, whereas MH cells became as excitable as LH cells. AP properties were similar between MH and LH cells, while ZI cells had a depolarized AP threshold. The higher AP threshold of ZI MCH neurons explains why these cells are less excitable than those in the other two regions. On the other hand, a modest difference in the firing response between MH and LH neurons may be due to some differences in active conductance that are yet to be investigated.

Another key finding of this study is that a subset of MCH neurons co-expressing CART have different electrophysiological properties compared to those devoid of CART. The previously described general electrophysiological properties of MCH neurons include a relatively hyperpolarized RMP, lack of spontaneous activity at rest, A-type current, spike adaptation upon injecting positive current, and absence of an H-current (Eggermann et al., 2003; Van Den Pol et al., 2004; Belanger-Willoughby et al., 2016). In our study, MCH/CART+ neurons displayed these aforementioned characteristics, consistent with previous studies. On the other hand, MCH/CART− neurons were relatively depolarized at rest and fired more APs upon stimulation with driving currents. As MCH/CART− neurons were primarily localized in the LH and sparsely elsewhere, their unique electrophysiological properties likely accounted for the area-dependent differences. Supporting this notion is our finding that the electrophysiological properties of MCH/CART+ neurons are largely the same across anatomical areas.

A striking feature specific to MCH/CART− neurons found in our study is the H-current. H-current is mediated by the hyperpolarization-activated cyclic nucleotide gated (HCN) channels, which are regulated by the second messenger cyclic AMP (cAMP) (Lüthi and McCormick, 1998; Wang et al., 2002). Thus, the presence of HCN channels provides a mechanism for neuromodulators acting via a cAMP-dependent pathway, such as adenosine and norepinephrine, to modulate this subpopulation of MCH neurons.

We also found that MCH/CART− neurons had a shorter AP half-width, which is consistent with a previous report (Fujita et al., 2021). Furthermore, another study found an intense spike adaptation in MCH/CART+ cells but not in MCH/CART− cells (Miller et al., 2024), which is in accordance with our finding that MCH/CART+ cells display a greater spike adaptation. However, this report did not find differences in the RMP of MCH/CART+ and MCH/CART− cells (Miller et al., 2024). This contrasts with our result showing significantly depolarized RMP of MCH/CART− neurons in the LH. The reasons for this discrepancy are unclear, but may include limiting the sampling of MCH/CART− neurons within the LH in our study, different chemical compositions of solutions used or housing conditions such as diet and room temperature.

Taken together, our study shows that MCH/CART− neurons in the LH are equipped with ionic mechanisms that allow them to be more excitable, including a depolarized RMP, H-current, and minimal spike adaptation. On the other hand, MCH/CART+ cells have largely similar electrophysiological properties, despite their relatively diffuse distribution across the hypothalamus and the zona incerta. This indicates that functional distinctions among these cells are more likely dependent on their extrinsic characteristics. It is worth noting that the hypothalamus receives inputs from diverse brain regions, some of which are spatially segregated, with the LH, MH and ZI receiving distinct range of inputs (Fujita et al., 2021; Wang et al., 2021). Conversely, MCH/CART+ and MCH/CART− neurons have different projection patterns (Cvetkovic et al., 2004). Specifically, MCH/CART+ neurons follow an ascending path to cortical areas, while MCH/CART− neurons project more caudally, innervating the brainstem and spinal cord. Thus, MCH neurons within different anatomical localization may constitute a unique functional subgroup with discrete connectivity within a broader network. Therefore, accurate reporting of the anatomical and/or neurochemical features of MCH neurons in future studies would enhance our understanding of the MCH system.

## Data Availability

The raw data supporting the conclusions of this article will be made available by the authors, without undue reservation.
