# Supplementary material for: Electrophysiological properties of melanin-concentrating hormone neuron subpopulations defined by anatomical localization and CART expression
Source: Front Cell Neurosci. 2025 Jan 22;18:1439752. doi: 10.3389/fncel.2024.1439752 (PMC11794810; doi:10.3389/fncel.2024.1439752)
Supplement: Supplementary file 1 [file Data_Sheet_1.PDF]

Supplementary information

Electrophysiological properties of melanin-concentrating hormone neuron subpopulations defined by anatomical localization and CART expression

Adekunle, Rafiat Damilola*; Chowdhury, Mohammed Sohel*; Fang, Lisa Z.; Hirasawa, Michiru

* Equal contribution

Supplementary Figure 1-5

Supplementary Table 1 (statistical information for Suppl Figures)

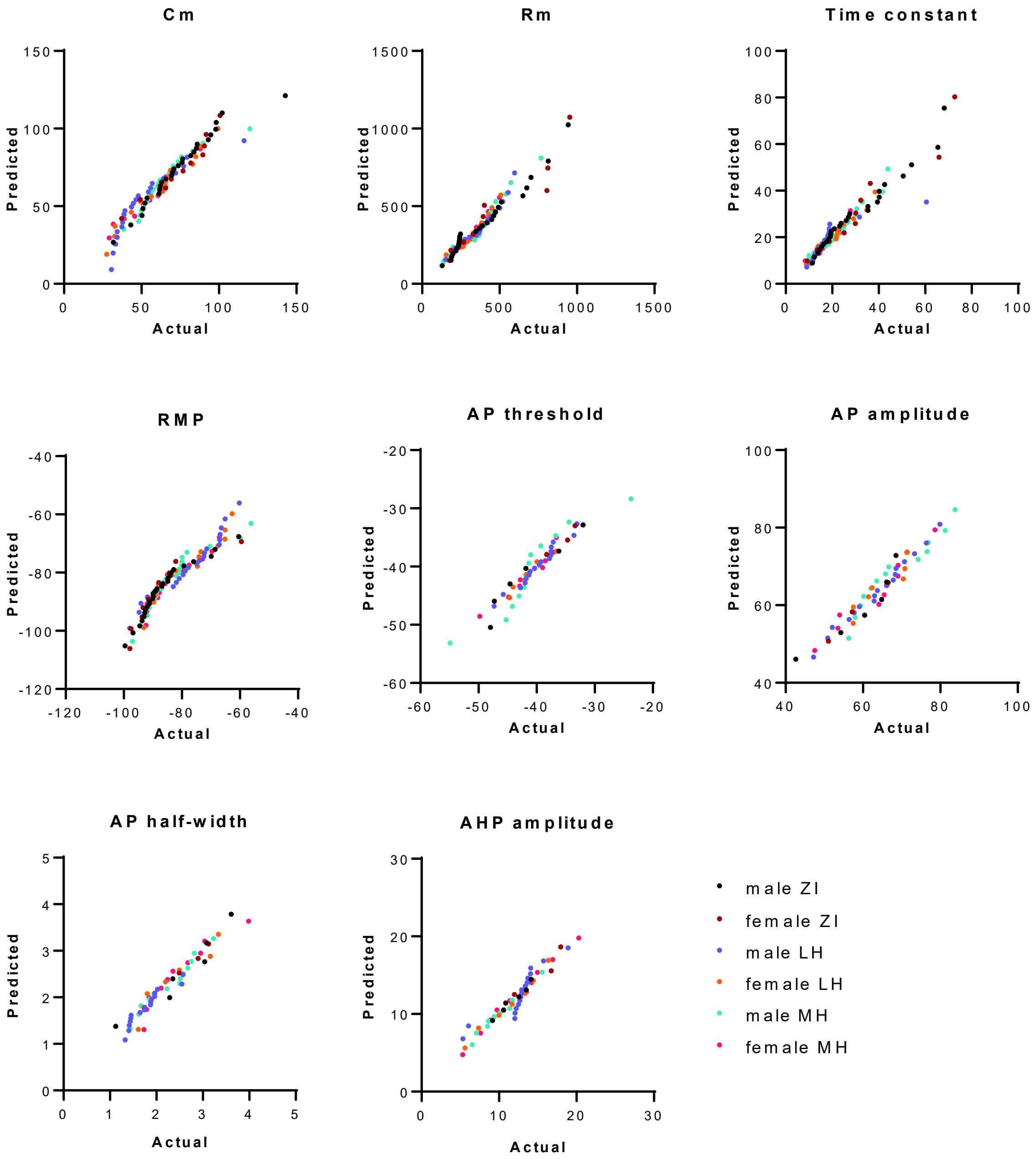

Supplementary Fig. 1: Quantile-quantile (Q-Q) plot of the distribution of indicated variables.
A theoretical normal distribution (A, D, F, H, G) or lognormal distribution (B, C) are depicted. Accompanies data shown in Figure 2.

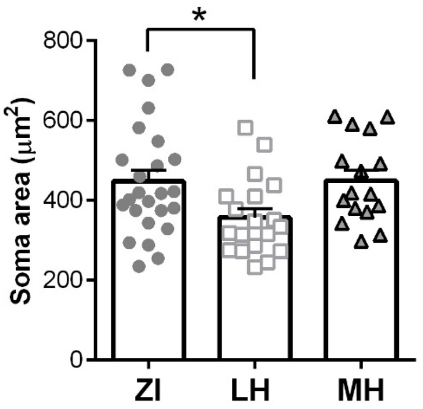

Supplementary Fig. 2: Somatic area of MCH neurons.
MCH neurons were filled with biocytin during patch clamp recording, then biocytin was visualized later by staining with AMCA-streptoavidin. Somatic area (excluding dendrites) was measured using ImageJ by two experimenters blind to the anatomical information of the cells. ZI: zona incerta, LH: lateral hypothalamus, MH: medial hypothalamus.

One-way ANOVA with Tukey's multiple comparison test. *$p<0.05$.
n = 16 – 25 cells /group.

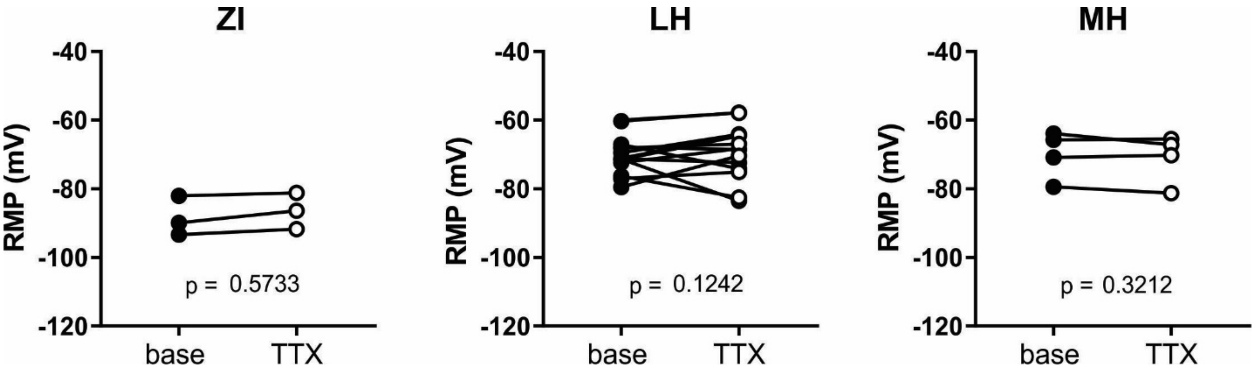

Supplementary Fig. 3: The resting membrane potential (RMP) of MCH neurons is insensitive to Na^+ channel blockade.
RMP was recorded before (base, closed circle) and after bath application of tetrodotoxin (TTX 1 μM; open circle).

No statistical difference was found by paired t-test in all three cell groups ($p>0.05$).
n = 3 – 13 cells/group.

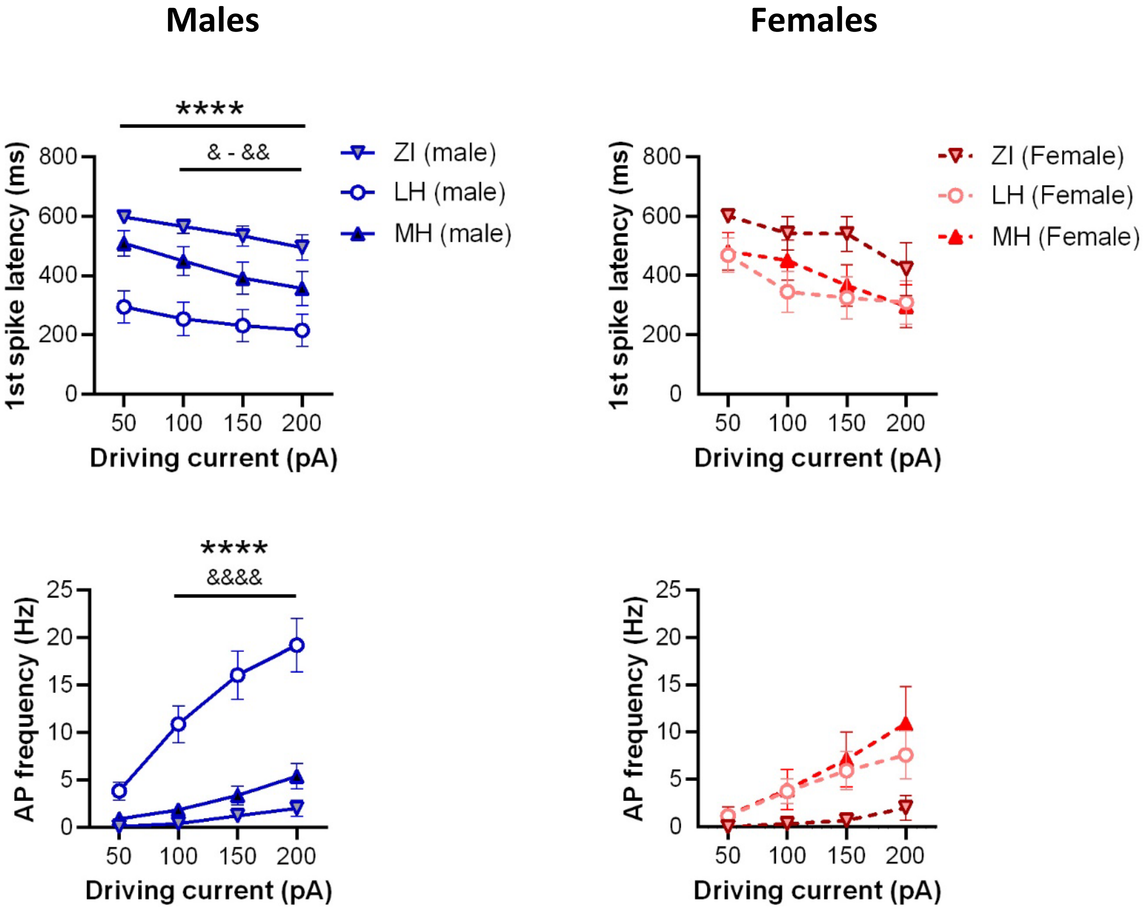

Supplementary Fig. 4: First spike latency and AP frequency of MCH neurons during a series of driving current injections in males (left) and females (right).
Accompanies data shown in Figure 3A and B.

Two-way ANOVA with Tukey's multiple comparison test.
LH vs. ZI: ****$p<0.0001$
LH vs. MH: $^{\&}p<0.05$, $^{\&\&}p<0.01$, $^{\&\&\&\&}p<0.0001$

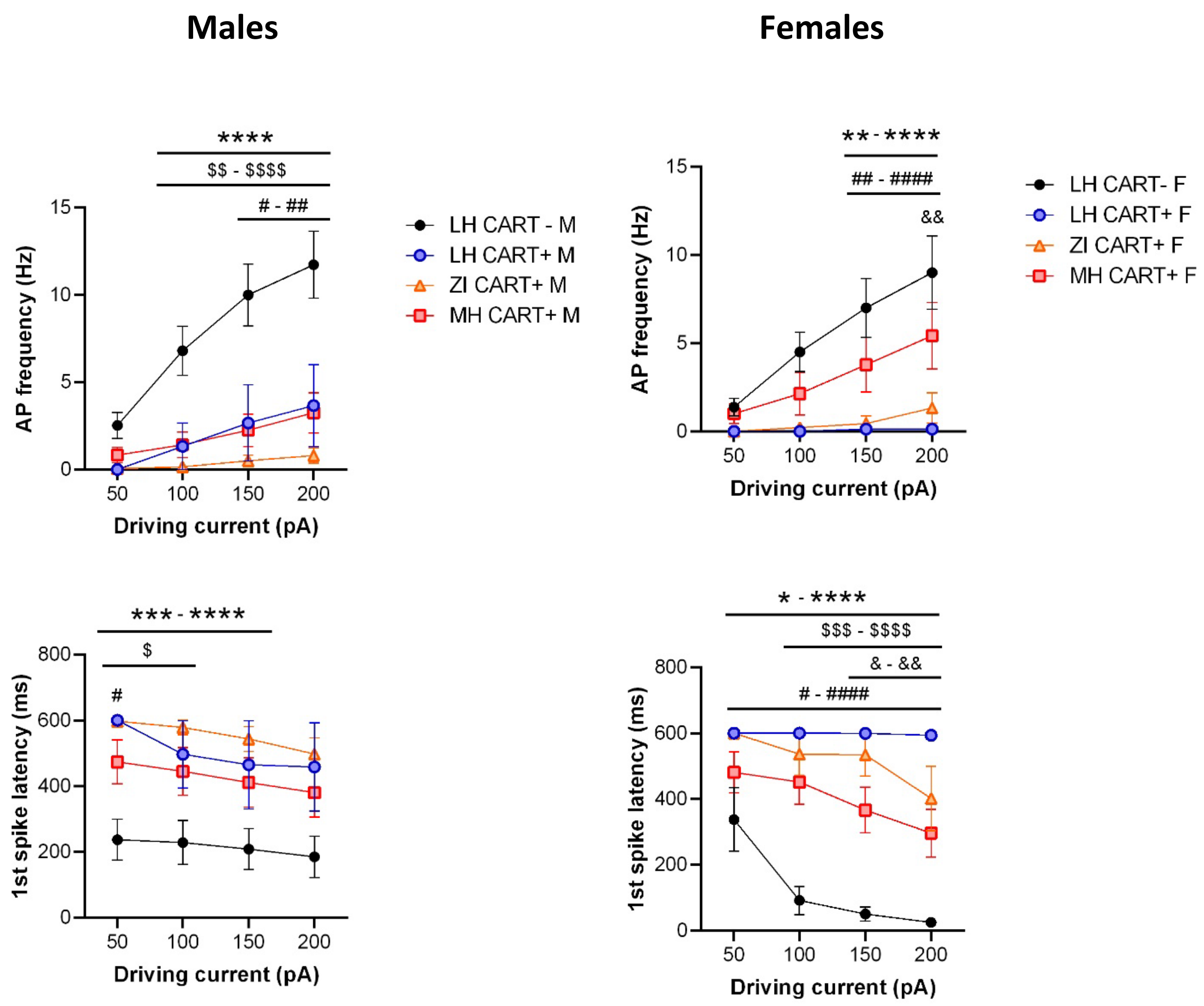

Supplementary Fig. 5: AP frequency and first spike latency of MCH neuron subpopulations during a series of driving current injections in males (M, left) and females (F, right).
Accompanies data shown in Figure 6F and G.

Two-way ANOVA with Tukey's multiple comparison test.
LH CART- vs ZI CART +: *p<0.05, **p<0.01, ***p<0.001, ****p<0.0001
LH CART- vs MH CART+: $^{\$}$p<0.05, $^{\$\$}$p<0.01, $^{\$\$\$}$p<0.001, $^{\$\$\$\$}$p<0.0001
LH CART- vs LH CART+: $^{\#}$p<0.05, $^{\#\#}$p<0.01, $^{\#\#\#\#}$p<0.0001
LH CART+ vs. MH CART+: $^{\&}$p<0.05, $^{\&\&}$p<0.01

Supplementary Table 1: Statistical information for Supplementary Figures.
Significant p values (p < 0.05) are bolded.

Sample size (cell/mice)	Test	P value	F, t
Supplementary Figure 2			
LH = 19/11<br>MH = 16/12<br>ZI = 25/17	1-way ANOVA	**0.0279**	F (2, 57) = 3.812
Supplementary Figure 3			
ZI = 3/2<br>LH = 12/4<br>MH = 4/1	Two tailed Paired t test	ZI p = 0.1242<br>LH p = 0.223<br>MH p = 0.3212	ZI t=2.566, df=2<br>LH t = 1.294, df = 11<br>MH t=1.185, df=3
Supplementary Figure 4			
Male ZI = 26/18<br>Male LH = 22/12<br>Male MH = 23/17	2 way ANOVA	1st spike latency<br>Interaction: 0.6316<br>Driving current: **<0.0001**<br>Area: **<0.0001**<br>AP freq<br>Interaction: **<0.0001**<br>Driving current: **<0.0001**<br>Area: **<0.0001**	Interaction: F (6,207) = 0.7229<br>Driving current : F(1.670, 115.2) = 16.13<br>Area: F (2, 69) = 13.83<br>Interaction: F (6, 204) = 19.85;<br>Driving current: F (1.231, 83.71) = 62.27<br>Area: F (2, 68) = 24.26
Female ZI = 10/7<br>Female LH = 16/7<br>Female MH = 14/9	2 way ANOVA	1st spike latency<br>Interaction: 0.4894<br>Driving current: **<0.0001**<br>Area: 0.1937<br>AP freq<br>Interaction: 0.3085<br>Driving current: **0.0003**<br>Area: 0.1624	Interaction: F (6, 111) = 0.9116<br>Driving current: F (1.807, 66.86) = 12.78<br>Area: F (2, 37) = 1.717<br>Interaction: F (6, 111) = 1.206 Driving current: F (1.221, 45.18) = 13.21<br>Area: F (2, 37) = 1.910
Supplementary Figure 5			
Male<br>LH CART- = 15/8<br>LH CART+ = 4/3<br>ZI CART+ = 20/13<br>MH CART+ = 12/8	2 WAY ANOVA	1st spike latency<br>Interaction: 0.9999<br>Driving Current: 0.2571<br>Cell group: **<0.0001**<br>AP freq<br>Interaction: **0.0022**<br>Driving Current: **0.0003**<br>Cell group: **<0.0001**	Interaction: F (9, 184) = 0.07590<br>Driving Current: F (3, 184) = 1.358<br>Area: F (3, 184) = 29.88<br>Interaction: F (9, 184) = 3.017<br>Driving Current: F (3, 184) = 6.514<br>Area: F (3, 184) = 47.12
Female<br>LH CART- = 8/4<br>LH CART+ = 8/3<br>ZI CART+ = 9/7<br>MH CART+ = 14/8	2 WAY ANOVA	1st spike latency<br>Interaction: 0.4682<br>Driving Current: **0.0019**<br>Cell group : **<0.0001**<br>AP freq<br>Interaction: 0.2531<br>Driving Current: **0.0013**<br>Cell group: **<0.0001**	Interaction: F (9, 140) = 0.9692<br>Driving Current: F (3, 140) = 5.211<br>Area: F (3, 140) = 36.27<br>Interaction: F (9, 140) = 1.280<br>Driving Current: F (3, 140) = 5.549<br>Area: F (3, 140) = 14.98
